# Challenges and Perspectives for Clinical Applications of Immersive and Non-Immersive Virtual Reality

**DOI:** 10.3390/jcm11154540

**Published:** 2022-08-04

**Authors:** Augusto Fusco, Gaetano Tieri

**Affiliations:** 1 UOC Neuroriabilitazione ad Alta Intensità, Fondazione Policlinico Universitario A. Gemelli IRCCS, 00168 Rome, Italy; 2 Virtual Reality Lab, University of Rome Unitelma Sapienza, Piazza Sassari, 4, 00161 Rome, Italy; 3 IRCCS Fondazione Santa Lucia, 00179 Rome, Italy

The development of rehabilitative technologies able to increase the intensity and the amount of time for daily treatment as well as the patients’ motivation and interest is a high-priority area of scientific research. So far, positive outcomes have been obtained by combining the rehabilitation and clinical protocols with different technologies, including robotics, assistive devices, neuroprostheses, brain–computer interface and even smartphone or tablet applications [1]. More recently, a growing amount of scientific evidence, coming from Neuroscience, Psychology, Medicine, Neurorehabilitation and Sport Rehabilitation, suggests that Virtual Reality (VR) may be an optimal solution for rehabilitation of different diseases. Indeed, due to its technical properties (i.e., high level of ecological validity, smart interface with other medical devices, 3D simulation of real-life experience, naturalistic interaction between user and virtual environment) and its strong effect on human perception and behaviour, VR is opening the way for implementing the next generation of cognitive/motor treatments and clinical applications [2].

However, despite the many efforts being made in this topic, a clear comprehension of VR’s efficacy in rehabilitation and clinical applications is still far off. One of the main problems stems from the improper use of the terminology in the literature, where the term “VR” is often used to describe the technologies that do not fully satisfy the VR specifications (i.e., serious games or videogames simply displayed on a monitor). Thus, it is important to better clarify the terminology to distinguish the two sides of VR technology, namely immersive and non-immersive VR. According to Slater [3], immersivity is determined by the number and range of a user’s sensory and motor channels connected to the system and is generated by combining different technologies in a whole system able to deliver visual information changed in real time according to the movement of the user’s head and body, as they would if he/she were in an equivalent physical environment [3,4]. Thus, while in a non-immersive VR system the virtual environment is displayed on a standard computer monitor and the interaction is limited to the use of a mouse, joystick or remote control, in an immersive VR system (typically constituted by new generation of head-mounted display or Cave Automatic Virtual Environment system, CAVE) the user is “surrounded by a 3D computer-generated representation” and can use their own body for a naturalistic sensory–motor interaction with the virtual environment.

Importantly, evidence highlights that the exposure to an immersive VR is able to elicit a sense of presence, i.e., a strong feeling of ‘being physically present’ in the virtual environment [4] which allows one to respond in a realistic way to the virtual stimuli and elicits physiological reactions as if the subject is physically situated in a real place [4,5,6]. Previous findings show that the sense of presence is a necessary mediator for eliciting real emotions when being in a virtual environment [5] and activating brain mechanisms underlying sensorimotor integration and the cerebral networks regulating focused attention [7]. Moreover, it has been shown that presence is elicited by fully immersive VR compared to less immersive 2D ones and, importantly, can impact the effectiveness of virtual treatments [8] and increase the degree to which the virtual stimuli are translated into real-world behaviour [9]. Interestingly, some studies exploited the advantage of sense of presence for realizing specific virtual experience for clinical applications. For example, Iosa et al. [10] induced in post-stroke patients the illusion of painting some art masterpieces for motor rehabilitation of the upper limb [10]. Furthermore, D’Antonio et al. [11] used an immersive VR treatment on balance and posture by inducing in stroke patients the illusion of movement (when they were still on a baropodometric platform inside a CAVE system) by showing a virtual environment programmed for movement in different directions at walking or running speed [11]. However, it is important to also highlight that non-immersive VR systems, such as serious games or 2D tasks, can have a potential impact on the rehabilitation process [12]. Indeed, these systems have been used in a wide range of health care applications, including both upper and lower limbs, orthopaedic rehabilitation and balance and gait training, with the aim of providing an intervention context for reacquisition, recovery or maintenance of motor and sensorial functions to ensure the quality of life of patients [13].

Thus, despite the technical and theoretical differences between immersive and non-immersive VR, both technologies can have different potential impacts for implementing novel rehabilitation treatments and allow scientists to optimize and personalize the experimental set-up according to the patient’s and hospital’s needs, including the possibility to develop tele-rehabilitation applications that patients can perform at home. The VR applications can be very versatile, using different systems and configurations, as well as different contents that can range from very engaging, dynamic and interactive to non-immersive and static [14]. These statements are also supported by the constant improvement of technology. Devices are becoming more portable and efficient with a reduction in costs, facilitating the ability to navigate and interact in real time with virtual environments enriched by specific sensory information [15]. So far, the VR clinical applications cover a wide range of areas including cognitive and motor rehabilitation in Parkinson’s [16] and Alzheimer’s disease [17], brain injury [18], phantom limb pain [19], cerebral palsy [20], unilateral spatial neglect [21,22], pain management [23], specific phobia [5] and eating disorders [24]. VR thus offers the opportunity to expand and improve the range of interventions for various rehabilitation applications. For example, it can provide real-time multisensory feedback, task variation and progression, and task-oriented repetitive training [25]. Regardless of the application, a large number of studies have shown how different treatments are able to influence functional outcomes as a function of dose and time of administration, particularly for rehabilitation. However, high therapeutic doses and patient adherence to the treatment plan are often limited [26]. VR can also improve the accuracy of performance measurements and the standardization of treatment protocols [2]. Furthermore, it can have positive effects on motivation. Physical and cognitive exercise is essential in increasing brain repair and plasticity [27]. Finally, VR could help to develop rehabilitation training ecologically, expanding the effects of training to activities of daily living and to the home [28]. However, the use of virtual reality still has many points to clarify and challenges to address. The wide application of VR still entails a small number of enrolled patients in several different diseases. Even if most of the data show improvements, patients enrolled in scientific protocols are still few. In some diseases, such as brain injury, this could also be due to severe conditions, which limit adherence to the rehabilitation program. The cognitive characteristics of patients can achieve the overall effectiveness of VR treatments, limiting the use in daily practice of neurological rehabilitation [29]. Moreover, VR treatment is highly dependent on the patient’s ability to use VR systems independently, in relation to the safety of use, the subject’s experience and usability of the devices [26]. Yet, there is still no clear evidence on the added value and cost-effectiveness of rehabilitation with VR compared to other therapies. Finally, the high sense of presence in the virtual environment can induce related pathologies (cybersickness), based on the features of the VR application and on the characteristics of the users undergoing treatment [30].

This Special Issue of the *Journal of Clinical Medicine* aims to collect works that shed new insights regarding the use of VR technologies, both immersive and non-immersive, for clinical and medical applications in order to provide novel evidence that could be useful for future development. Our proposal is to add scientific data to implement research in rehabilitation with VR. Additional scientific data and reviews on peculiar issues of VR are needed to test its effectiveness in rehabilitation training on a larger population sample for various levels of disability and in domestic contexts after hospital discharge. Finally, we would like to include in the manuscript a final brief section entitled “Implications of using Virtual Reality technology in Clinical Practice” in order to give practical information for using the proposed method and findings that could help clinicians and rehabilitators for an optimal application in clinical practice.

## References

[1] Frontera W.R., Bean J.F., Damiano D., Ehrlich-Jones L., Fried-Oken M., Jette A., Jung R., Lieber R.L., Malec J.F., Mueller M.J. (2017). Rehabilitation research at the National Institutes of Health: Moving the field forward (executive summary). Neurorehabilit. Neural Repair.

[2] Tieri G., Morone G., Paolucci S., Iosa M. (2018). Virtual reality in cognitive and motor rehabilitation: Facts, fiction and fallacies. Expert Rev. Med. Devices.

[3] Slater M. (1999). Measuring Presence: A Response to the Witmer and Singer Presence Questionnaire. Presence Teleoperators Virtual Environ..

[4] Sanchez-Vives M.V., Slater M. (2005). From presence to consciousness through virtual reality. Nat. Rev. Neurosci..

[5] Parsons T.D., Rizzo A.A. (2008). Affective outcomes of virtual reality exposure therapy for anxiety and specific phobias: A meta-analysis. J. Behav. Ther. Exp. Psychiatry.

[6] Jelić A., Tieri G., De Matteis F., Babiloni F., Vecchiato G. (2016). The Enactive Approach to Architectural Experience: A Neurophysiological Perspective on Embodiment, Motivation, and Affordances. Front. Psychol..

[7] Vecchiato G., Tieri G., Jelic A., De Matteis F., Maglione A.G., Babiloni F. (2015). Electroencephalographic Correlates of Sensorimotor Integration and Embodiment during the Appreciation of Virtual Architectural Environments. Front. Psychol..

[8] Villani D., Riva F., Riva G. (2007). New technologies for relaxation: The role of presence. Int. J. Stress Manag..

[9] Persky S., Blascovich J. (2008). Immersive virtual video game play and presence: Influences on aggressive feelings and behavior. Presence Teleoperators Virtual Environ..

[10] Iosa M., Aydin M., Candelise C., Coda N., Morone G., Antonucci G., Marinozzi F., Bini F., Paolucci S., Tieri G. (2021). The Michelangelo Effect: Art Improves the Performance in a Virtual Reality Task Developed for Upper Limb Neurorehabilitation. Front. Psychol..

[11] D’Antonio E., Tieri G., Patané F., Morone G., Iosa M. (2020). Stable or able? Effect of virtual reality stimulation on static balance of post-stroke patients and healthy subjects. Hum. Mov. Sci..

[12] Vieira C., Pais-Vieira C.F.D.S., Novais J., Perrotta A. (2021). Serious Game Design and Clinical Improvement in Physical Rehabilitation: Systematic Review. JMIR Serious Games.

[13] Laamarti F., Eid M., El Saddik A. (2014). An overview of serious games. Int. J. Comput. Games Technol..

[14] Yeung A.W.K., Tosevska A., Klager E., Eibensteiner F., Laxar D., Stoyanov J., Glisic M., Zeiner S., Kulnik S.T., Crutzen R. (2021). Virtual and Augmented Reality Applications in Medicine: Analysis of the Scientific Literature. J. Med. Internet Res..

[15] Slater M., Sanchez-Vives M.V. (2016). Enhancing Our Lives with Immersive Virtual Reality. Front. Robot. AI.

[16] dos Santos Mendes F.A., Pompeu J.E., Lobo A.M., da Silva K.G., de Paula Oliveira T., Zomignani A.P., Piemonte M.E.P. (2012). Motor learning, retention and transfer after virtual-reality-based training in Parkinson’s disease–effect of motor and cognitive demands of games: A longitudinal, controlled clinical study. Physiotherapy.

[17] Foloppe D.A., Richard P., Yamaguchi T., Etcharry-Bouyx F., Allain P. (2018). The potential of virtual reality-based training to enhance the functional autonomy of Alzheimer’s disease patients in cooking activities: A single case study. Neuropsychol. Rehabil..

[18] Rose F.D., Brooks B.M., Rizzo A.A. (2005). Virtual Reality in Brain Damage Rehabilitation: Review. Cyberpsychol. Behav..

[19] Murray C.D., Patchick E.L., Caillette F., Howard T., Pettifer S. (2006). Can immersive virtual reality reduce phantom limb pain?. Stud. Health Technol. Inform..

[20] Ravi D., Kumar N., Singhi P. (2017). Effectiveness of virtual reality rehabilitation for children and adolescents with cerebral palsy: An updated evidence-based systematic review. Physiotherapy.

[21] Ogourtsova T., Silva W.S., Archambault P.S., Lamontagne A. (2017). Virtual reality treatment and assessments for post-stroke unilateral spatial neglect: A systematic literature review. Neuropsychol. Rehabil..

[22] Castiello U., Lusher D., Burton C., Glover S., Disler P. (2004). Improving left hemispatial neglect using virtual reality. Neurology.

[23] Dascal J., Reid M., IsHak W.W., Spiegel B., Recacho J., Rosen B., Danovitch I. (2017). Virtual Reality and Medical Inpatients: A Systematic Review of Randomized, Controlled Trials. Innov. Clin. Neurosci..

[24] Provenzano L., Porciello G., Ciccarone S., Lenggenhager B., Tieri G., Marucci M., Dazzi F., Loriedo C., Bufalari I. (2019). Characterizing Body Image Distortion and Bodily Self-Plasticity in Anorexia Nervosa via Visuo-Tactile Stimulation in Virtual Reality. J. Clin. Med..

[25] Porras D.C., Siemonsma P., Inzelberg R., Zeilig G., Plotnik M. (2018). Advantages of virtual reality in the rehabilitation of balance and gait: Systematic review. Neurology.

[26] Huygelier H., Mattheus E., Abeele V.V., Van Ee R., Gillebert C.R. (2021). The Use of the Term Virtual Reality in Post-Stroke Rehabilitation: A Scoping Review and Commentary. Psychol. Belg..

[27] Sale A., Berardi N., Maffei L. (2009). Enrich the environment to empower the brain. Trends Neurosci..

[28] Rizzo A.A., Schultheis M., Kerns K.A., Mateer C. (2004). Analysis of assets for virtual reality applications in neuropsychology. Neuropsychol. Rehabil..

[29] Fusco A., Giovannini S., Castelli L., Coraci D., Gatto D.M., Reale G., Pastorino R., Padua L. (2022). Virtual Reality and Lower Limb Rehabilitation: Effects on Motor and Cognitive Outcome—A Crossover Pilot Study. J. Clin. Med..

[30] Weech S., Kenny S., Barnett-Cowan M. (2019). Presence and Cybersickness in Virtual Reality Are Negatively Related: A Review. Front. Psychol..

